# Supplementary material for: A Fetal Brain magnetic resonance Acquisition Numerical phantom (FaBiAN)
Source: Sci Rep. 2022 May 23;12:8682. doi: 10.1038/s41598-022-10335-4 (PMC9127105; doi:10.1038/s41598-022-10335-4)
Supplement: Supplementary file 1 — Supplementary Information. [file 41598_2022_10335_MOESM1_ESM.pdf]

Supplementary Information - A Fetal Brain magnetic resonance Acquisition Numerical phantom (FaBiAN)

Hélène Lajous[1,2,*], Christopher W. Roy[1,+], Tom Hilbert[1,3,4,+], Priscille de Dumast[1,2], Sébastien Tourbier[1], Yasser Alemán-Gómez[1], Jérôme Yerly[1,2], Thomas Yu[4], Hamza Kebiri[1,2], Kelly Payette[5,6], Jean-Baptiste Ledoux[1,2], Reto Meuli[1], Patric Hagmann[1], Andras Jakab[5,6], Vincent Dunet[1], Mériam Koob[1], Tobias Kober[1,3,4,§], Matthias Stuber[1,2,§], and Meritxell Bach Cuadra[2,1]

[1]Department of Radiology, Lausanne University Hospital (CHUV) and University of Lausanne (UNIL), Lausanne, Switzerland
[2]CIBM Center for Biomedical Imaging, Switzerland
[3]Advanced Clinical Imaging Technology (ACIT), Siemens Healthcare, Lausanne, Switzerland
[4]Signal Processing Laboratory 5 (LTS5), Ecole Polytechnique Fédérale de Lausanne (EPFL), Lausanne, Switzerland
[5]Center for MR Research, University Children's Hospital Zurich, University of Zurich, Zurich, Switzerland
[6]Neuroscience Center Zurich, University of Zurich, Zurich, Switzerland
[*]helene.lajous@unil.ch
[+,§]These authors contributed equally to this work.

Supplementary Methods

Supplementary Method S1: Extended Phase Graphs (EPG) formalism

The EPG concept combines two approaches to efficiently account for the evolution of a significant ensemble of isochromats, and therefore accurately characterize echoes (type, intensity, timing): the configuration states and the partition state[1,2]. The magnetization is decomposed in the Fourier domain in so-called "configuration states". In this representation, the dephasing action of gradients on magnetization translates to dephasing the order of the configuration states. RF pulses then allow isochromats to pass from a configuration state to another of same order, in agreement with the partition state method that describes the magnetization after an RF pulse as split into three components: the dephasing transverse magnetization, the rephasing transverse magnetization from which an echo may arise, and the longitudinal magnetization. Figure S1 provides an EPG diagram of the simulated FSE sequences.

Supplementary Method S2: Motion index computation

The motion index we introduce in this study was originally developed in the framework of the MIAL Super-Resolution Toolkit[4]. The motion index is estimated from tracking the displacement of the centroids of the 2D brain masks. It is computed as the sum of the variances of the 2D brain mask centroid coordinates of adjacent slices over the central third of the 3D brain mask, normalized by the number of slices considered:

$$index = \frac{Var(centroid_x) + Var(centroid_y)}{nb\_slices}$$

where $centroid_x$, respectively $centroid_y$, is the vector of x, respectively y, coordinates of the 2D brain mask centroid of each slice over the central third of the 3D brain mask, and nb_slices is the number of slices considered.

The code to compute the motion index can be found here: http://github.com/Medical-Image-Analysis-Laboratory/mialsuperresolutiontoolkit/blob/e02b7a2/pymialsrtk/interfaces/preprocess.py#L949-L1280.

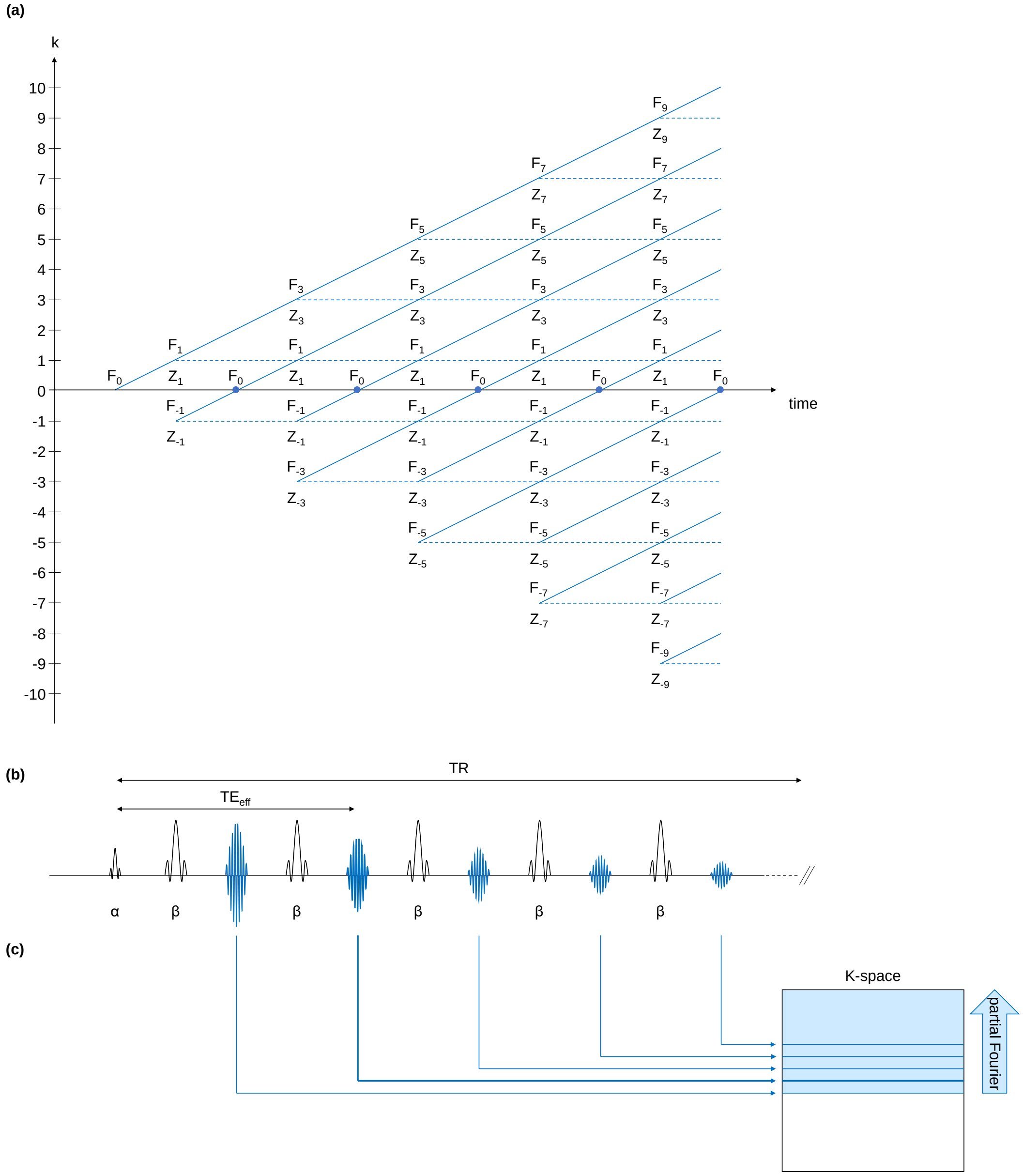

Figure S1. (a) EPG diagram of a fast spin echo (FSE) sequence. A first RF pulse ($\alpha = 90^{\circ}$) excites the magnetization to form a free induction decay. The following RF refocusing pulses ($120^{\circ} \leq \beta \leq 180^{\circ}$) split it in agreement with the partition state method, and generate spin and stimulated echoes. The longitudinal magnetization is depicted in dashed lines, its transverse component in solid lines respectively. $F_{+}(k)$ denotes the dephasing transverse magnetization, $F_{-}(k)$ the rephrasing transverse magnetization, its pendant that is responsible for the measured echoes. Z represents the longitudinal magnetization. Adapted from the literature[1,2]. (b) Sequence pulse design of a FSE sequence. The effective echo time (TEeff) corresponds to the time after the RF excitation pulse at which the echo that will encode the central line of K-space is measured. In clinical practice, the angle of the RF refocusing pulses can vary between 120° and 180° to account for strict SAR limits in fetal imaging. In our simulations, the echo train length is equal to 224 and the echo spacing, which is the time interval between two successive echoes, ranges between $4.08ms$ and $10ms$. Adapted from the literature[3]. (c) K-space sampling. Each echo allows to sample a line in k-space, the echo occurring at the effective echo time corresponding to the central line of k-space. Slightly more than half of k-space is actually sampled from the information of the 224 echoes in the echo train. The remaining part of k-space is recovered using the properties of Hermitian symmetry in the Fourier domain. Thus, a complete slice is encoded during one repetition time (TR).

Supplementary Figures

Figure S2 displays the histograms of the distribution of (a) gestational age (from 21 to 35 weeks, 27.8 ± 4.40 weeks) across the original clinical cases and simulated subjects involved in the data augmentation experiment (Application 2), and (b) in-plane isotropic resolution in the corresponding low-resolution SS-FSE images of the fetal brain according to the main magnetic field strength.

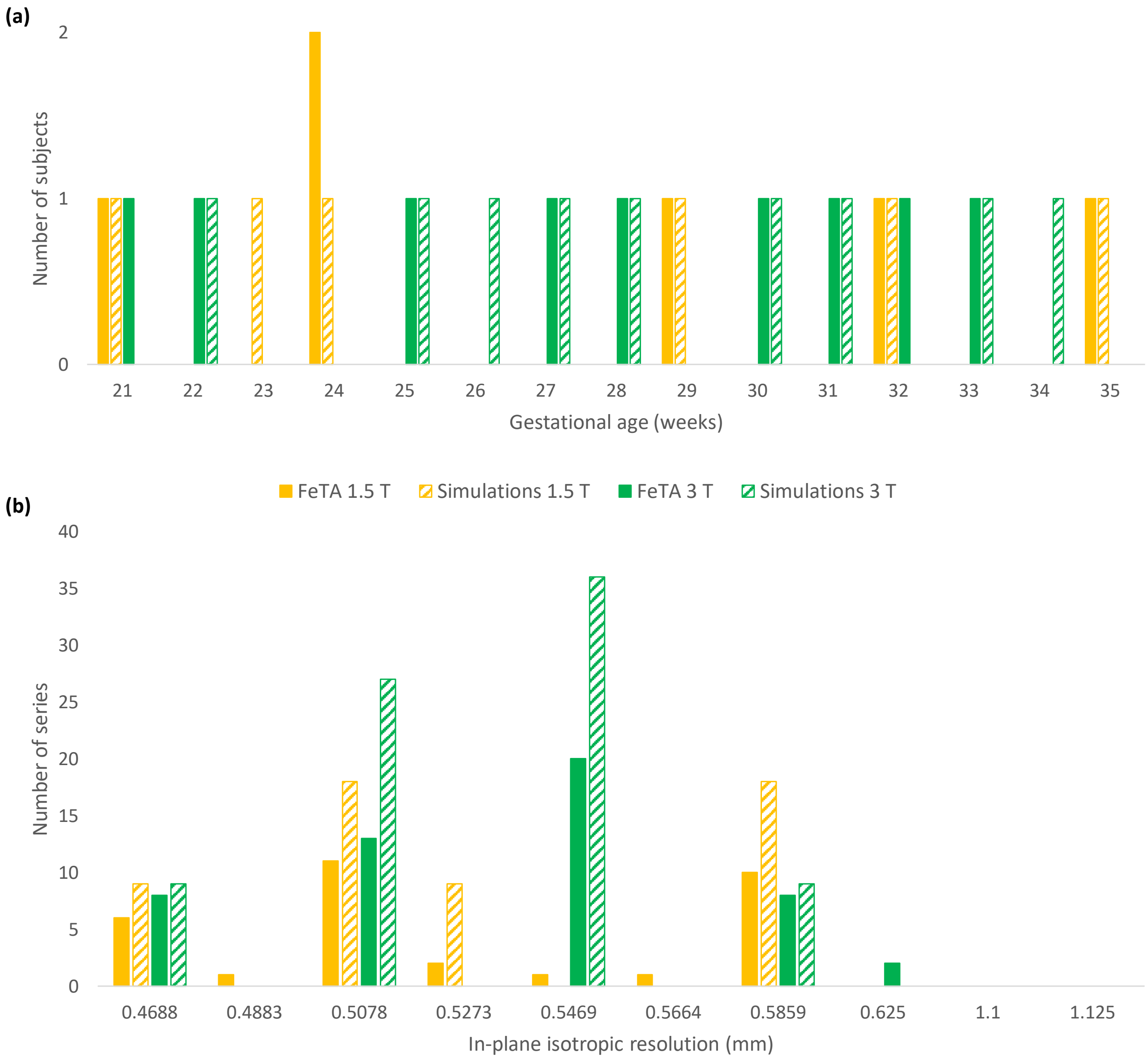

Figure S2. Distribution of (a) gestational age across all clinical cases and simulated subjects involved in the data augmentation experiment and (b) in-plane isotropic resolution in the corresponding low-resolution SS-FSE images of the fetal brain according to the main magnetic field strength.

Figure S3 shows the close resemblance of SS-FSE images, whether acquired/simulated at 1.5 T or 3 T.

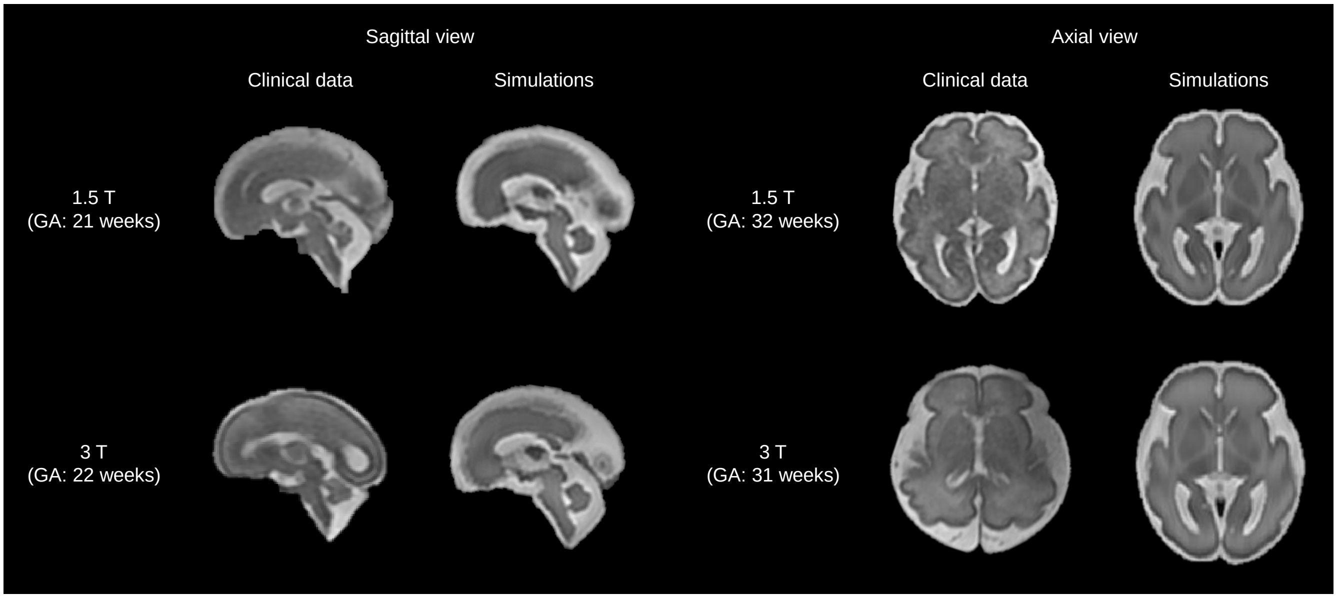

Figure S3. Visual inspection and comparison of SS-FSE images of the fetal brain acquired/simulated at 1.5 T or 3 T.

Supplementary Table

Table S1 provides further details on the ranges of values that are relevant to an application in fetal brain MRI, thus consistent with common clinical protocols, and that were tested during the development of the first FaBiAN prototype[5].

	Range
GA (weeks)	18 – 38
Magnetic field strength	1.5 T or 3 T
Field inhomogeneities	from low to strong
Acquisition parameters	
Contrast	
Effective echo time (*ms*)	86 – 431
Echo spacing (*ms*)	4.08 – 10
Echo train length	134 – 224
Excitation flip angle (°)	90
Refocusing pulse flip angle (°)	120 – 180
Geometry	
Slice thickness (*mm*)	1 – 4
Slice gap (*mm*)	0 – 0.4
Number of slices	37 – 51
Phase oversampling (%)	0 – 80
Shift of the field-of-view (*mm*)	± 2
Resolution	
Field-of-view (mm^2)	$240 \times 240 - 360 \times 360$
Base resolution (*voxels*)	256 – 327
Phase resolution (%)	70 – 100
Reconstruction matrix	$256 \times 179 - 512 \times 512$
Amplitude of 3D rigid motion	
Translation (*mm*) in x	± 10
Translation (*mm*) in y	± 10
Translation (*mm*) in z	± 10
3D rotation (°)	± 10
Noise	
Mean	0
Standard deviation	0.01 – 0.3

Table S1. Clinically relevant ranges of values for fast spin echo (FSE) sequences in fetal brain MRI. These different acquisition parameters and settings were tested during the development of the first FaBiAN prototype.

References

1. Busse, R. F., Hariharan, H., Vu, A. & Brittain, J. H. Fast spin echo sequences with very long echo trains: Design of variable refocusing flip angle schedules and generation of clinical t2 contrast. *Magn. Reson. Medicine* **55**, 1030–1037, DOI: https://doi.org/10.1002/mrm.20863 (2006).

2. Weigel, M. Extended phase graphs: Dephasing, RF pulses, and echoes - pure and simple. *J. Magn. Reson. Imaging* **41**, 266–295, DOI: https://doi.org/10.1002/jmri.24619 (2015).

3. Patel, M. R., Klufas, R. A., Alberico, R. A. & Edelman, R. R. Half-fourier acquisition single-shot turbo spin-echo (HASTE) MR: Comparison with fast spin-echo MR in diseases of the brain. *Am. J. Neuroradiol.* **18**, 1635–1640 (1997).

4. Tourbier, S., De Dumast, P., Kebiri, H., Hagmann, P. & Bach Cuadra, M. Medical-Image-Analysis-Laboratory/mialsuperresolutiontoolkit: MIAL Super-Resolution Toolkit v2.0.1. *Zenodo*, DOI: https://doi.org/10.5281/zenodo.4392788 (2020).

5. Lajous, H., Roy, C. W., Yerly, J. & Bach Cuadra, M. FaBiAN v1.2, DOI: https://doi.org/10.5281/zenodo.5471094 (2022).
